# Obituary Gerhart Drews

**DOI:** 10.1007/s00203-023-03568-y

**Published:** 2023-05-15

**Authors:** Georg Fuchs, Rolf Thauer

**Affiliations:** 1 Freiburg, Germany; 2 Marburg, Germany


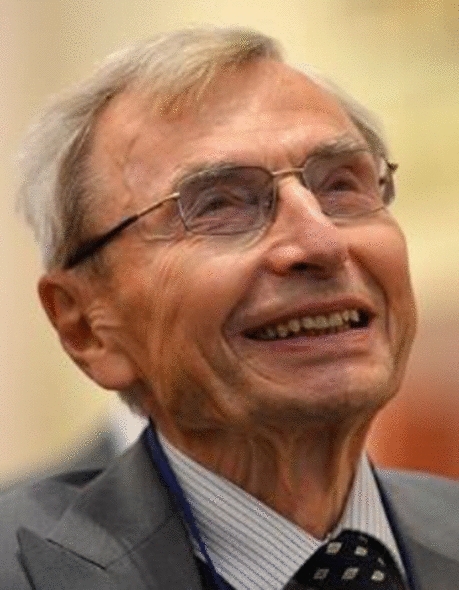


The Freiburg microbiologist Prof. em. Dr rer. nat. Dr h. c. Gerhart Drews died on April 6, 2023; on May 30, 2023 he would have been 98 years old. To the very end, he remained well-read, full of enterprising spirit and devoted to his friends with empathy. With him we lose a fascinating personality who shaped scientific microbiology in the field of phototrophic bacteria.

Gerhart Drews’ life began in 1925 in Berlin-Charlottenburg as the son of a family of teachers. He lived in Berlin until he graduated from high school in 1943 and was shaped by the cultural life of the capital during this time. His lifelong enthusiasm for drama and opera has its roots here. After graduating from high school, military service and imprisonment followed, from which he was released in 1945. His older brother fell in World War II.

From 1946 he studied biology, geography and chemistry for teaching in Halle. There Kurt Mothes (biochemistry) and above all Johannes Buder (botany) inspired him. After the state examination in 1951, Drews did his doctoral thesis under the guidance of Buder on phototaxis in filamentous cyanobacteria, in which he discovered different dependencies of topotaxis and phobotaxis on the wavelength and intensity of light. His colleague at that time was Hans Günter Schlegel, co-editor of Archives of Microbiology for decades.

After receiving his doctorate in 1953, Gerhart Drews worked as a postdoc at the Institute for Microbiology and Experimental Therapy of the Academy of Sciences in Jena on the microbial polyphosphate metabolism and at the same time began investigations into the ultrastructure and cytochemistry of phototrophic bacteria. His investigations made a significant contribution to the discovery of the intracytoplasmic membrane systems (thylakoids) in cyanobacteria and purple bacteria. With this work, Gerhart Drews habilitated in Halle in 1960. In the same year he married Christiane May, who at the time was senior physician for gynecology at the University Clinic in Halle and with whom he remained in love until her death at the end of 2021.

Although both held secure positions and he was offered a professorship in Rostock, they secretly fled the increasingly repressive, communistic German Democratic Republic in early 1961 on separate paths. Gerhard Drews accepted an offer for a position at the Faculty of Biology at the University of Freiburg. When he received a call to the Chair of Microbiology in Tübingen in 1963, he preferred the Freiburg counter-offer. Here he founded the scientific microbiology and worked from 1964 to 1993 as a professor.

Drews’ research focus remained the biology of phototrophic bacteria. He was particularly fascinated by these organisms as model systems for research into cell differentiation. His working group, which at times included e.g. Jürgen Oelze, Georg Schön, Jürgen Weckesser, Jochen Golecki and Gabriele Klug soon formed a leading international center for bacterial photosynthesis research, which attracted a large number of foreign scientists. Several study visits to the USA, e.g. with Howard Gest and Martin Kamen, deepened the international cooperation. Together with Norbert Pfennig and Rogier Stanier he organized in 1970 the first international “Symposium on Photosynthetic Prokaryotes” in Freiburg.

Gerhart Drews was co-editor of Archives of Microbiology for decades and for many years chaired the selection committee for biology at the Deutsche Forschungsgemeinschaft and was a member of the selection committee for the Humboldt Foundation. In 1999 he received the Heisenberg Medal for his extraordinary commitment.

“But what remains?” It is his many pioneering works in the field of bacterial photosynthesis and cell biology; his book “Molecular Plant Virology” (2004); but also his essays on Anton de Bary, a Freiburg mycologist, and on Ferdinand Cohn, the patron of Robert Koch; and the memory of a wise, humble, dependable friend and colleague. Anyone who did not know Gerhart Drews personally can get a vivid picture of him from his autobiography “Reflektionen eines Biologen auf seine Zeit.” Verlag Dr. Kovac, Hamburg (2002).

